# Effects of thoracic radiotherapy timing and duration on progression‐free survival in limited‐stage small cell lung cancer

**DOI:** 10.1002/cam4.1616

**Published:** 2018-07-17

**Authors:** Shen Zhao, Ting Zhou, Shuxiang Ma, Yuanyuan Zhao, Jianhua Zhan, Wenfeng Fang, Yunpeng Yang, Xue Hou, Zhonghan Zhang, Gang Chen, Yaxiong Zhang, Yan Huang, Li Zhang

**Affiliations:** ^1^ Zhongshan School of Medicine Sun Yat‐Sen University Guangzhou China; ^2^ Department of Medical Oncology Sun Yat‐Sen University Cancer Center Guangzhou China; ^3^ State Key Laboratory of Oncology in South China Guangzhou China; ^4^ Collaborative Innovation Center for Cancer Medicine Guangzhou China

**Keywords:** concurrent chemoradiotherapy, limited‐stage small cell lung cancer, progression‐free survival, thoracic radiotherapy duration, thoracic radiotherapy timing

## Abstract

Concurrent chemoradiotherapy (CRT) has been recommended and applied widely as the standard treatment for limited‐stage small cell lung cancer (LS‐SCLC). However, controversies remain regarding the optimal timing and treatment duration of thoracic radiotherapy (TRT), and their effects on patient survival. To evaluate prognostic values of TRT timing and duration on progression‐free survival (PFS) in LS‐SCLC and their dependence on TRT fractionation and clinicopathological characteristics, we retrospectively analyzed 197 LS‐SCLC patients receiving CRT from 2000 to 2016 at Sun Yat‐sen University Cancer Center. Based on the optimal cut‐off values of TRT timing and duration generated by Cutoff Finder, patients were divided into early TRT/late TRT group and short TRT/long TRT group respectively. Univariate and multivariate Cox analysis were performed to assess correlations of TRT timing, duration, fractionation, and clinicopathological characteristics with PFS. Univariate analysis revealed that early‐initiated TRT (*P *= 2.54 × 10^−4^) and short TRT (*P *= .001) significantly correlated with longer PFS. Their PFS benefits persisted in patients receiving hyperfractionated TRT and etoposide‐cisplatin (EP) chemotherapy, but were less prominent in those receiving once‐daily TRT and non‐EP chemotherapy. Multivariate analysis further identified early initiated TRT (*P *= .004) and short TRT (*P *= .017) as independent prognostic factors for longer PFS in LS‐SCLC. Our study confirmed that early‐initiated TRT and short TRT had positive prognostic roles in LS‐SCLC, especially in patients receiving hyperfractionated TRT and etoposide‐cisplatin chemotherapy. TRT fractionation was not an independent prognostic factor in LS‐SCLC.

## 1 INTRODUCTION

Lung cancer is the leading cause of cancer‐related deaths in the world.^1^ Small cell lung cancer (SCLC), the most aggressive subtype characterized by high progression rate and metastatic risk, accounts for 15% of all diagnosed cases.^2^ Based on the Veteran Affairs Lung Study Group (VALSG) staging system, SCLC is typically staged into limited‐stage (LS) and extensive‐stage (ES), each accounting for 40% and 60% respectively.^3^, ^4^

According to the American College of Chest Physicians guidelines, standard first‐line treatment for LS‐SCLC is concurrent chemoradiotherapy (CRT), adopting accelerated hyperfractionated (twice‐daily) thoracic radiotherapy (TRT) and concurrently‐delivered etoposide + cisplatin (EP)‐based chemotherapy.^5^, ^6^ However, many controversies remain regarding the optimized design of CRT, especially the TRT timing, duration, and fractionation. Several clinical trials attempting to address this problem reported inconsistent results.^7^, ^8^, ^9^, ^10^ At least 6 meta‐analyses^11^, ^12^, ^13^, ^14^, ^15^, ^16^ also tried to reconcile this problem. Most of the meta‐analyses confirmed the survival benefits of early TRT,^11^, ^12^, ^13^, ^15^, ^16^ but some argued that benefits of early TRT are limited to patients receiving twice‐daily TRT only.^12^ Some studies observed significant correlations between overall treatment duration and overall survival (OS),^13^, ^14^ suggesting that overall treatment duration may be a potential prognostic factor in LS‐SCLC.

Therefore, to evaluate prognostic values of TRT timing and duration in LS‐SCLC and to investigate whether their effects are dependent on TRT fractionation or certain clinicopathological characteristics, we performed a retrospective study of LS‐SCLC patients receiving CRT at our center here. To avoid confounding effects of subsequent treatments, progression‐free survival (PFS), instead of OS, was used as the primary endpoint in this study.^13^, ^17^

## 2 MATERIALS AND METHODS

### 2.1 Patients

A total of 754 patients diagnosed with LS‐SCLC consecutively between October 2000 and May 2016 at Sun Yat‐sen University Cancer Center (SYSUCC) were identified. The inclusion criteria were: (1) aged 18 years or older, (2) have histologically or cytologically confirmed small‐cell lung cancer, (3) had received chemotherapy followed by TRT, and (4) had complete information regarding TRT timing, duration, fractionation, chemotherapy regimen, and pretreatment albumin levels. Among the 754 patients, 538 who did not receive TRT or received radiotherapy limited to mediastinum and supraclavicular lymph nodes were excluded. Another 19 patients were also excluded because they received TRT before chemotherapy. Finally, a total of 197 patients were eligible and enrolled in this study. All patients had provided inform consent before the initiation of chemotherapy.

### 2.2 Data extraction

Patient baseline characteristics including age, gender, smoking status, comorbidities, and pretreatment albumin level were extracted using a standard data extraction system. Comorbidity scores were calculated based on the Charlson Comorbidity Index^18^ before the initiation of chemotherapy. Information regarding chemotherapy and TRT were extracted from the electronic medical records. TRT timing was calculated as the time interval between the first day of chemotherapy and the first day of TRT. TRT duration and fractionation of each patient were directly extracted from their radiotherapy records.

### 2.3 Follow‐up

All patients were regularly followed up after the day of pathological diagnosis. During the follow‐up, patients received computed tomography (CT) scans after every 2 cycles of chemotherapy or every 6 weeks. Disease progression (PD) was evaluated by systematic radiologic review committee based on the Response Evaluation Criteria in Solid Tumors (RECIST 1.0). PFS was defined as the time elapsed from the date of pathological diagnosis to the date of CT scan that confirmed PD. The last date of follow‐up was November 30, 2014. Patients who did not have PD at that time were censored.

### 2.4 Statistical analysis

Categorical variables were compared using Chi‐Square or Fisher's exact tests and were represented as numbers or percentage of patients. Continuous variables were dichotomized into categorical variables at their median values. As a result of the inconsistent definitions of early TRT and short TRT in previous studies, the optimal cut‐off points of TRT timing and duration were determined by web‐based R software‐engineered system, Cutoff Finder (http://molpath.charite.de/cutoff/).^19^ All patients were divided into early TRT group, late TRT group, short TRT group, and long TRT group based on the corresponding cut‐off points. Univariate analysis adopted the Kaplan‐Meier method to assess prognostic effects of TRT timing and duration on PFS in the whole cohort and in subgroups stratified by RT fractionations. Significant differences were detected using log‐rank tests. Multivariate analysis was performed using the Cox‐proportional hazard model. Results with a two‐sided *P* value <.05 were deemed statistically significant. All statistical analyses were performed using the SPSS version 21 (IBM Inc., Armonk, NY) software.

## 3 RESULTS

### 3.1 Patient characteristics

The median age of enrolled patients was 53 years (range, 32‐87 years). The majority of them were males (n = 172, 87.31%), smokers (n = 144, 73.10%) and had a comorbidity score of 0 (n = 162, 82.23%). In terms of treatments, all patients received platinum‐based chemotherapy as the first‐line treatment, most of which was etoposide ‐cisplatin (n = 175, 88.83%). 126 of 197 patients (63.96%) received hyperfractionated twice‐daily TRT, while the rest (n = 71, 36.04%) received once‐daily TRT. The median pretreatment albumin level of all patients was 41.90 g/L (range, 24.46‐50.70 g/L).

### 3.2 Patient categorization based on cut‐off values

Based on the optimal cutoff value of TRT timing defined by Cutoff Finder, patients were divided into early TRT group (TRT timing ≤96 days: n = 135, 68.53%) and late TRT group (TRT timing>96 days: n = 62, 31.47%) respectively. Similarly, patients were divided into short TRT group (TRT duration ≤31 days: n = 122, 61.93%) and long TRT group (TRT duration > 31 days: n = 75, 38.07%) based on the optimal cut‐off value of TRT duration determined by Cutoff Finder.

### 3.3 Association of TRT timing and duration with clinicopathological characteristics

Patient clinicopathological characteristics were summarized based on TRT timing and duration respectively (Tables 1 and 2). Compared with patients receiving late TRT, patients receiving early TRT were significantly younger (*P *=* *.014) and more likely to receive hyperfractionated twice‐daily TRT (*P *=* *2.51 × 10^−5^). Shorter TRT duration also significantly correlated with younger age (*P *=* *.001) and higher probability of receiving hyperfractionated TRT (*P *=* *.009). Other patient characteristics including gender, smoking status, comorbidity score, chemotherapy regimen, and pretreatment albumin level were comparable between patients with different TRT timings and patients with different TRT durations.

**Table 1:** Patient characteristics stratified based on TRT timing (n = 197)

Variables	Early TRT		Late TRT		*P* value^a^
	No.	%	No.	%	
Age (y)					
≤53	81	60.00	25	40.32	.014^b^
>53	54	40.00	37	59.68	
Gender					
Male	119	88.15	53	85.48	.647
Female	16	11.85	9	14.52	
Smoking					
Current/ever	99	73.33	45	72.58	1.000
Never	36	26.67	17	27.42	
Comorbidity score					
=0	122	84.14	50	80.65	.692
≥1	23	15.86	12	19.35	
Chemotherapy regimen					
EP	117	86.67	58	93.55	.223
Others	18	13.33	4	6.45	
RT fractionation					
Once daily	35	25.93	36	58.06	2.51 × 10^−5^ b
Twice daily	100	74.07	26	41.94	
Albumin					
Relatively low	62	45.93	34	54.84	.284
Relatively high	73	54.07	28	45.16	

EP, etoposide‐platinum; RT, radiotherapy; TRT, thoracic radiotherapy.

a Log‐rank test.

b Statistically significant.

**Table 2:** Patient characteristics stratified based on TRT duration (n = 197)

Variables	Short TRT		Long TRT		*P* value^a^
	No.	%	No.	%	
Age (y)					
≤53	77	63.11	29	38.67	.001^b^
>53	45	36.89	46	61.33	
Gender					
Male	105	86.07	67	89.33	.660
Female	17	13.93	8	10.67	
Smoking					
Current/ever	89	72.95	55	73.33	1.000
Never	33	27.05	20	26.67	
Comorbidity score					
=0	99	81.15	63	84.00	.703
≥1	23	18.85	12	16.00	
Chemotherapy regimen					
EP	105	49.30	67	89.33	1.000
Others	108	50.70	8	10.67	
RT fractionation					
Once daily	35	28.69	36	48.00	.009^b^
Twice daily	87	71.31	39	52.00	
Albumin					
Relatively low	59	48.36	37	49.33	1.000
Relatively high	63	51.64	38		

EP, etoposide‐platinum; RT, radiotherapy; TRT, thoracic radiotherapy.

a Log‐rank test.

b Statistically significant.

### 3.4 Association of TRT timing and duration with PFS

**Figure 1:**
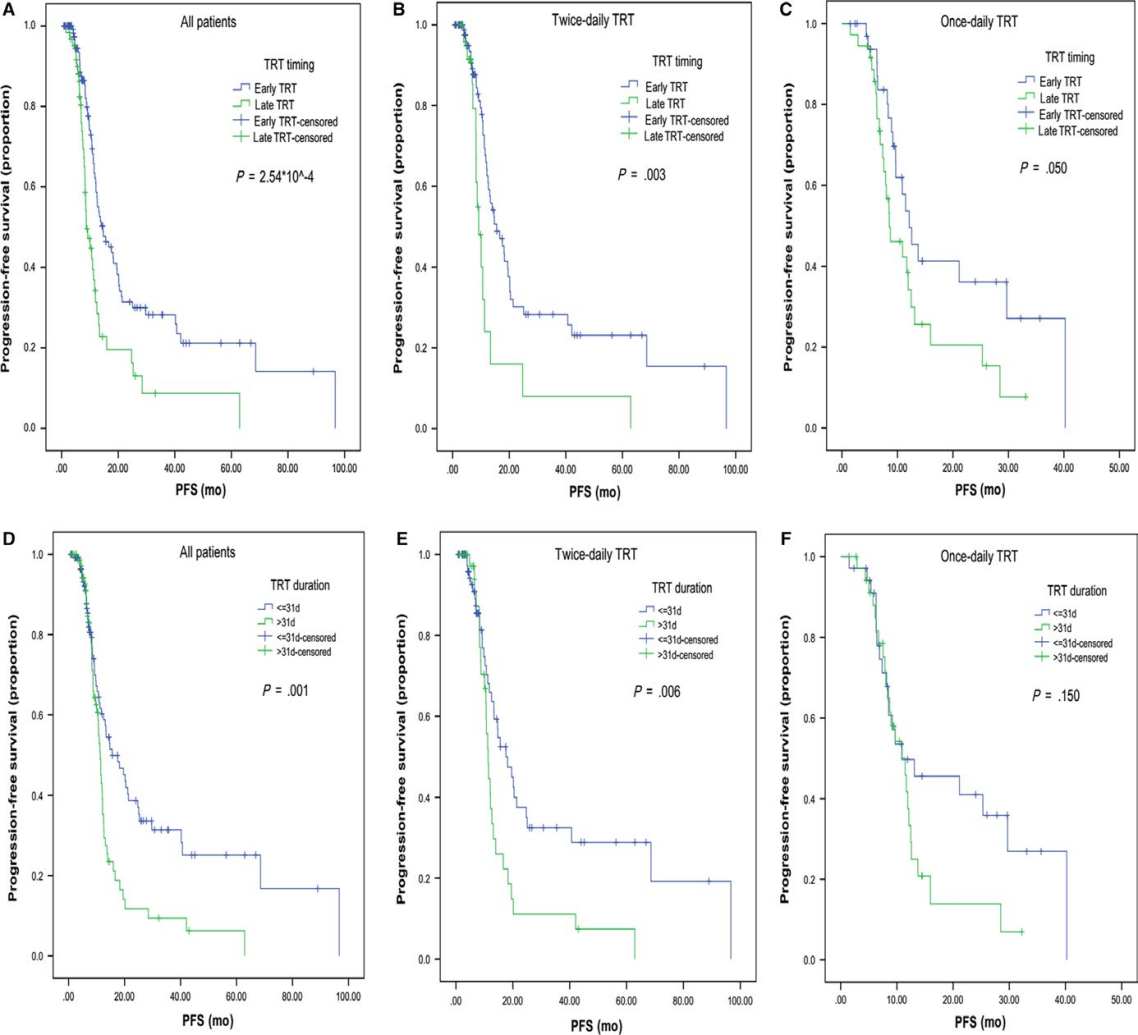
Kaplan Meier survival curves comparing PFS. A‐C, Early TRT vs Late TRT; A, Overall patients. B, Patients who received twice‐daily TRT. C, Patients who received once‐daily TRT. D‐E, Short TRT vs Long TRT; D, Overall patients. E, Patients who received twice‐daily TRT. F, Patients who received once‐daily TRT

Median PFS of enrolled patients was 12.13 months (range: 0.77‐96.70 months). Results of univariate analyses revealed that early initiated TRT significantly correlated with longer PFS in comparison to late‐initiated TRT (Figure 1A: 14.63 vs 8.73 months, *P *=* *2.54 × 10^−4^). The difference remained significant in patients receiving twice‐daily TRT (Figure 1B: 15.57 vs 9.23 months *P *=* *.003), while marginally significant in those receiving once‐daily TRT (Figure 1C: 12.13 vs 8.53, *P *=* *.050). Likewise, short TRT demonstrated significant PFS advantages over long TRT in all enrolled patients (Figure 1D: 15.57 vs 11.30 months, *P *=* *.001) and in patients receiving hyperfractionated TRT (Figure 1E: 17.57 vs 11.30 months, *P *=* *.006). No significant PFS benefit of short TRT was observed in patients treated with once‐daily TRT (Figure 1F: 10.93 vs 10.87 months, *P *=* *.150).

Univariate analyses of TRT fractionation and clinicopathological characteristics revealed that hyperfractionated twice‐daily TRT showed significant PFS benefits based on Breslow test (13.33 vs 10.93 months, *P *=* *.041), but not the log‐rank test (*P *=* *.100). No significant correlation between PFS and age (*P *=* *.491), gender (*P *=* *.796), smoking status (*P *=* *.389), comorbidity score (*P *=* *.992), chemotherapy regimen (*P *=* *.250), and pretreatment albumin level (*P *=* *.639) was observed.

Prognostic effects of TRT timing, duration and fractionation on PFS were validated in the multivariate analysis (Table 3). Both TRT timing and duration appeared to be independent prognostic factors for PFS. In comparison to early TRT, late TRT showed 1.835‐fold higher risk of progression (HR = 1.835; 95%CI: 1.211‐2.781; *P *=* *.004), while long TRT significantly correlated with 1.643‐fold higher risk of progression comparing to short TRT (HR = 1.643; 95%CI: 1.094‐2.467; *P *=* *.017). In the multivariate analysis, RT fractionation was not identified as an independent prognostic factor for PFS (*P *=* *.631).

**Table 3:** Cox regression model^a^ analysis

Variables	Hazard ratio	95% CI		*P* value
		LL	UL	
TRT timing				
Early TRT	1.000	—	—	.004^b^
Late TRT	1.835	1.211	2.781	
TRT duration				
Short <=31 d	1.000	—	—	.017^b^
Long >31 d	1.643	1.094	2.467	
RT fractionation				
Once daily	—	—	—	.631
Twice daily	—	—	—	

CI, confidence interval; LL, lower limit; RT, radiotherapy; TRT, thoracic radiotherapy; UL upper limit.

a Adjusted for TRT timing, RT duration and RT fractionation.

b Statistically significant.

**Figure 2:**
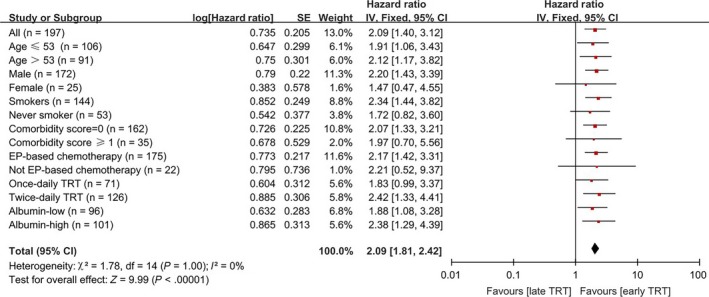
Forest plot depicting PFS based on subgroup analysis between Early and Late TRT regimens. Data are derived from Cox's analysis without covariates

**Figure 3:**
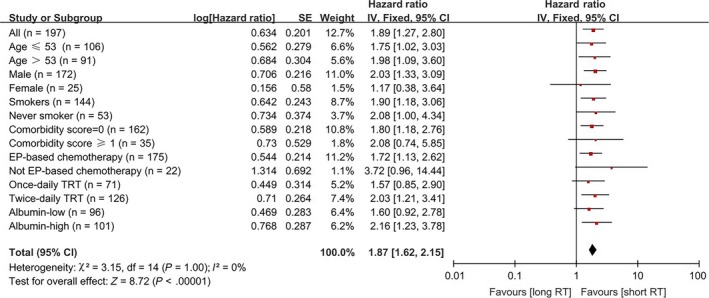
Forest plot depicting PFS based on subgroup analysis between Short and Long TRT regimens. Data are derived from Cox's analysis without covariates

Prognostic values of TRT timing and duration for PFS were further evaluated in subgroup patients with different clinicopathological characteristics. All subgroup analysis results had hazard ratios (HR) >1, suggesting consistent PFS benefits of early and short TRT in patients carrying different characteristics. Specifically, early TRT linked with significantly longer PFS in patients from all age groups, males, smokers, patients with comorbidity score = 0, and any pretreatment albumin levels (Figure 2). While short TRT significantly correlated with longer PFS in patients of any age, males, smokers and never‐smokers, patients with comorbidity score = 0, and relatively high pretreatment albumin levels (Figure 3). Of note, in patients treated with EP‐ based chemotherapy and twice‐daily TRT, early and short TRT both showed significant PFS benefits.

## 4 DISCUSSION

In this study, we retrospectively analyzed 197 LS‐SCLC patients treated with CRT at SYSUCC, aiming to evaluate prognostic impacts of TRT timing and duration on PFS and whether these impacts are dependent on TRT fractionation or certain clinicopathological characteristics in LS‐SCLC. Our multivariate analysis identified early TRT initiation and short TRT duration as independent prognostic factors for longer PFS in LS‐SCLC, significantly predicting 5.90‐month and 4.27‐month PFS improvements respectively. When stratifying patients by RT fractionations and chemotherapy regimen, PFS benefits brought by early TRT and short TRT were more prominent in patients receiving hyperfractionated twice‐daily TRT and EP‐based chemotherapy, but not less in those receiving once‐daily TRT and non‐EP chemotherapy. In subgroup analyses based on baseline characteristics, early TRT and short TRT showed similar tendencies in predicting longer PFS across all subgroups.

Consistent with the preponderance of evidence, results of our study suggested that early TRT initiation significantly and independently correlated with longer PFS and could serve as an independent prognostic factor for better outcomes in LS‐SCLC. Previously, multiple randomized trials^7^, ^8^, ^9^, ^10^ and meta‐analyses^11^, ^12^, ^13^, ^14^, ^15^, ^16^ have assessed prognostic effects of TRT timing, duration, and fractionation on OS and PFS in LS‐SCLC. However, no unanimous conclusion has been reached thus far. Although the majority of studies supported survival benefits of early TRT, some argued that benefits of early TRT were dependent on TRT fractionation or chemotherapy regimen, and therefore TRT fractionation and apposite chemotherapy were more essential for patients’ survival.^16^, ^18^, ^19^, ^20^ However, our subgroup analysis results showed that PFS benefits brought by early TRT initiation, though were less prominent, still exist in patients receiving once‐daily TRT and non‐EP chemotherapy. In addition, Fried et al^12^ also detected modest but significant OS improvements in early TRT, regardless of chemotherapy regimen and RT fractionation. Another reason supporting early‐initiated TRT is that more cycles of chemotherapy preceding TRT may lead to increased toxicity and poor patient compliance,^21^ adversely affecting patient outcomes consequently. In summary, our results confirm that early‐initiated TRT independently correlated with longer PFS and is a potential prognostic factor for better outcomes in LS‐SCLC.

As for TRT duration, previous studies have argued that overall treatment time was more important than TRT timing for patient outcomes.^13^, ^14^, ^15^ Pijls‐Johannesma et al^14^ reported that patients who completed CRT within 30 days had significantly higher 5‐year survival rate than those who didn't. De Ruysscher et al^15^ also observed a significant correlation between higher 5‐year survival rate and shorter SER (the Start of any treatment until the End of Radiotherapy). In accordance with the above reports, our study found that shorter TRT duration was an independent prognostic factor for longer PFS in LS‐SCLC. The correlation between shorter TRT duration and longer PFS persisted in patients with different clinicopathological characteristics and were more prominent in those treated with hyperfractionated twice‐daily TRT and EP‐based chemotherapy. The prognostic role of TRT duration in LS‐SCLC may be explained by the treatment‐related repopulation, which has been confirmed as one of the main reasons for treatment failure and subsequent progression in LS‐SCLC.^22^, ^23^, ^24^, ^25^, ^26^, ^27^, ^28^, ^29^, ^30^ Compared with the overall treatment time (the period between the initiation of chemotherapy to the cessation of radiotherapy), it is more intuitive and applicable for radiologist to consider TRT timing and duration in clinical practice. Therefore, we concluded that early TRT initiation and short TRT duration are both independent prognostic factors for longer PFS in LS‐SCLC and should be equally emphasized when designing a CRT plan.

In terms of TRT fractionation and chemotherapy regimen, our study found that PFS benefits of early‐initiated TRT and short TRT were more prominent when combining with hyperfractionated TRT and EP‐based standard chemotherapy.

In contrary with the study by Wong et al,^17^ TRT fractionation was not identified as an independent prognostic factor for PFS in our study. Wong et al concluded that TRT fractionation was more important than TRT timing for survival in LS‐SCLC. However, their study did not incorporate TRT duration. Given the role of accelerated repopulation in LS‐SCLC recurrence and progression,^20^, ^22^, ^23^, ^24^, ^25^, ^26^, ^31^, ^32^ the prognostic effect of hyperfractionated TRT detected by Wong et al may be attributed to shorten TRT duration. In our multivariate analysis, which incorporated TRT timing, duration, and fractionation, only TRT timing and duration were identified as independent prognostic factors for PFS. Our subgroup analysis results further supported this finding by showing modest PFS benefits of early TRT initiation and short TRT duration in patients receiving once‐daily TRT. Based on current evidence, we concluded TRT fractionation was not an independent prognostic factor in LS‐SCLC. Nevertheless, large‐scale perspective studies are warranted to verify our findings.

EP‐based chemotherapy is the standard of care in LS‐SCLC.^5^, ^20^, ^31^ All patients enrolled in our study were treated with platinum‐based chemotherapy, and 88.83% of them received etoposide‐cisplatin. As the proportion of patients receiving non‐EP chemotherapy was too small, we failed to detect significant associations between chemotherapy regimen and PFS (*P *=* *.250). To address this issue, we performed subgroup analyses to evaluate impacts of TRT timing and duration on PFS in patients receiving EP and non‐EP chemotherapies respectively. In accordance with previous reports,^10^, ^14^, ^15^, ^32^ our results further confirmed the significance of apposite and uncompromising chemotherapy in LS‐SCLC. Based on our analysis (Figures 2 and 3), we concluded that appropriate systemic chemotherapy must be ensured before TRT timing and duration can make a real difference in survival outcomes.

We acknowledge that there are several limitations of our study. Firstly, it's a retrospective single‐centered study. To minimize potential selection bias, all patients were identified consecutively from October 2000 to May 2016. To mitigate the effect of small sample size and to ensure the credibility of our conclusions, subgroup analyses were performed to assess the consistency of TRT timing and duration as prognostic factors in patients with different clinicopathological characteristics. Secondly, we were unable to account for potential confounding factors in this study. For instance, patients with younger age and receiving hyperfractionated TRT may have better performance status or smaller tumor burdens, either of which can contribute to better outcomes. Therefore, large‐scale prospective studies are needed to further validate our findings.

## 5 CONCLUSIONS

Overall, our results suggest that early TRT initiation and short TRT duration are both independent prognostic factors for longer PFS in LS‐SCLC. Their PFS benefits are consistent in patients with different clinicopathological characteristics and more prominent in those receiving EP‐based standard chemotherapy and hyperfractionated TRT. Both TRT timing and duration should be equally emphasized in a CRT plan. However, early‐initiated TRT and short TRT duration also mean more intense and aggressive treatments that may lead to higher risk of adverse events. Therefore, future studies are warranted to further elucidate clinical implications of TRT timing and duration in LS‐SCLC.

## 6 CLINICAL PRACTICE POINTS

Concurrent chemoradiotherapy is the standard treatment for patients with limited‐stage small cell lung cancer (LS‐SCLC). Although the preponderance of evidence supports the delivery of early initiated thoracic radiotherapy (TRT), controversy remains regarding the role of TRT duration, fractionation, and the relative importance of the 3 factors. In this study, we analyzed data of 197 patients retrospectively to evaluate prognostic roles of TRT timing, duration, and fractionation for patients with LS‐SCLC. We found that early TRT initiation and short TRT duration were both independent prognostic factors for longer PFS. Their PFS benefits were consistent in patients with different clinicopathological characteristics, and more prominent in those receiving EP‐based standard chemotherapy and hyperfractionated TRT. In conclusion, this study suggested that both TRT timing and duration should be equally emphasized in the treatment of LS‐SCLC to achieve more favorable outcomes.

## CONFLICTS OF INTEREST

Authors declare that there are no conflicts of interest.
